# Antimalarial Activity of *Cocos nucifera* Husk Fibre: Further Studies

**DOI:** 10.1155/2013/742476

**Published:** 2013-07-30

**Authors:** J. O. Adebayo, E. A. Balogun, S. O. Malomo, A. O. Soladoye, L. A. Olatunji, O. M. Kolawole, O. S. Oguntoye, A. S. Babatunde, O. B. Akinola, A. C. C. Aguiar, I. M. Andrade, N. B. Souza, A. U. Krettli

**Affiliations:** ^1^Department of Biochemistry, University of Ilorin, Ilorin, Nigeria; ^2^Department of Physiology, University of Ilorin, Ilorin, Nigeria; ^3^Department of Microbiology, University of Ilorin, Ilorin, Nigeria; ^4^Department of Chemistry, University of Ilorin, Ilorin, Nigeria; ^5^Department of Hematology, University of Ilorin, Ilorin, Nigeria; ^6^Department of Anatomy, University of Ilorin, Ilorin, Nigeria; ^7^Programa de Pós Graduação em Medicina Molecular, Faculdade de Medicina, Universidade Federal de Minas Gerais, Belo Horizonte, 30130-100 MG, Brazil; ^8^Laboratório de Malária, Centro de Pesquisas René Rachou, FIOCRUZ, Belo Horizonte, 30130-100 MG, Brazil

## Abstract

In this study, the antimalarial and toxicity potentials of husk fibre extracts of five Nigerian varieties of *Cocos nucifera* were evaluated *in vitro*. The only active extract fraction, West African Tall (WAT) ethyl acetate extract fraction, was then evaluated for its phytochemical constituents, antimalarial and toxicity potentials at varying doses (31.25–500 mg/kg body weight) using various organ function indices. The results revealed that WAT ethyl acetate extract fraction (WATEAEF) contained alkaloids, tannins, and flavonoids and was active against *Plasmodium falciparum* W2 strain maintained in continuous culture, with a selectivity index of 30.3. The same extract fraction was active *in vivo* against *Plasmodium berghei* NK65, causing more than 50% reduction in parasitaemia on days 4 and 6 after inoculation at various doses administered. WATEAEF did not significantly alter (*P* > 0.05) function indices of the liver and cardiovascular system at all doses administered but significantly increased (*P* < 0.05) plasma creatinine concentration at 250 and 500 mg/Kg body weight compared to controls. The results of this study suggest that WATEAEF possesses antimalarial activity and may not adversely affect normal liver function nor predispose subjects to cardiovascular diseases but may impair normal kidney function at higher doses. Further studies are underway to isolate the active principles.

## 1. Introduction

Malaria is one of the most dreaded human parasitic diseases in the tropics and subtropics, especially in Africa where 81% of cases and 91% of deaths have been estimated to occur, with children under five years of age and pregnant women being most severely affected [1]. Nigeria accounts for a quarter of all malaria cases in Africa [2], mostly caused by *Plasmodium falciparum *[3], with an estimated 100 million malaria cases and over 300,000 deaths per year [4]. In addition to its direct health impact, malaria imposes a huge economic burden on afflicted individuals and nations, through high healthcare cost, missed days at work, and reduced economic output and productivity [5]. 

The continuous spread of *P. falciparum* resistance to antimalarial drugs poses a serious threat to malaria control programs. This, in addition to the high cost of the potent antimalarial drugs, has left the poor masses to be heavily reliant on traditional herbal medicines, which are often affordable and available [3]. Thus, the use of plant remedies has steadily increased worldwide in recent years, as well as the search for new phytochemicals that could be developed as useful drugs for the treatment of malaria and other infectious diseases [6]. *Cocos nucifera* husk fibre and white flesh are used in folk medicine for the treatment of malaria [3, 7]. Our recent *in vitro* studies have authenticated the acclaimed antimalarial action of the husk fibre extract of *Cocos nucifera* [8]. The present study was, therefore, set out to evaluate the antimalarial and toxicity potentials of the husk fibre extracts of five Nigerian varieties of *Cocos nucifera in vitro* and the most active *in vivo*, with the aim of identifying the most potent variety for rational antimalarial drug design.

## 2. Materials and Methods

### 2.1. Chemicals

Absolute n-hexane, ethyl acetate, methanol, and ethanol were obtained from Sigma-Aldrich Laborchemikalien GmbH, Germany. Giemsa stain was obtained from Anosantec Laboratory, UK. Sodium chloride and glucose were both obtained from British Drug House Chemical limited, Poole, England. Sodium citrate was obtained from Merck, Darmstadt, Germany. Disodium hydrogen phosphate and potassium dihydrogen phosphate were obtained from Kermel Chemicals, China. Immersion oil was obtained from Panzonar laboratory Supplies, Button road, Canada. RPMI 1640 medium, sodium bicarbonate, L-glutamine, D-sorbitol, and HEPES were obtained from Sigma (St. Louis, MO, USA). Other reagents were of analytical grade and were prepared with injection water.

### 2.2. Animals

Sixty-five adult Swiss albino mice with an average weight of 18 ± 2 g were obtained from the Animal Breeding Unit of the Faculty of Pharmacy, Obafemi Awolowo University, Ile Ife, Osun State, Nigeria. 

### 2.3. Plant Materials

Husk fibres of five varieties of *Cocos nucifera* dried at room temperature under shade were obtained from Nigeria Institute for Oil Palm Research (NIFOR), Badagry, Lagos State, Nigeria, in October 2010. They include West African Tall, Dwarf Red, Dwarf Yellow, Dwarf Green, and Hybrid varieties. They were botanically authenticated at the institute by Mr. Igbene Collins. 

### 2.4. Parasite Strains

A chloroquine sensitive strain of *Plasmodium berghei *(NK-65) was obtained from the Department of Pharmacology, Obafemi Awolowo University, Ile-Ife, Nigeria. The W2 clone, CQ-resistant and mefloquine-sensitive strain of *P. falciparum* was originally received from the New York University Medical School and the *in vitro* tests were performed at Laboratório de Malária, Centro de Pesquisas René Rachou, FIOCRUZ, Belo Horizonte, MG, Brazil.

### 2.5. Preparation of Extracts

The extracts were prepared according to the method of Adebayo et al. [9]. The fresh samples were allowed to dry under shade at room temperature and then pulverized into powder. Four hundred and fifty grams (450 g) of the powder of each variety was successively extracted with 5000 mL each of n-hexane, ethyl acetate, and methanol for 72 h per solvent in a tightly stoppered glass container. After each solvent extraction, the content was filtered with Whatman filter paper no. 1. The filtrates were then concentrated under pressure using rotary evaporator at 40°C, thereby generating the crude extracts.

### 2.6. Qualitative Phytochemical Screening

Phytochemical screening was carried out for the ethyl acetate extract fraction using standard procedures described by Sofowora [10] to evaluate the presence of tannins, anthraquinones, alkaloids, terpenes, saponins, flavonoids, phenols, steroids, and cardiac glycoside.

### 2.7. *In Vitro* Studies

#### 2.7.1. [^3^H]-Hypoxanthine Incorporation Assay

The *in vitro* tests were performed with blood parasites of *P. falciparum* W2 clone, which are chloroquine resistant, kept in continuous culture at 37°C in human erythrocytes, using the candle jar method as described by Trager and Jensen [11]. The antiplasmodial effects of the extract fractions were measured through inhibition of parasite growth, by the [^3^H]-hypoxanthine incorporation assay, as described by Desjardins et al. [12] and modified by Zalis et al. [13]. For the test, a stock solution of each extract fraction was diluted in complete culture medium without hypoxanthine (RPMI 1640 containing 10% human serum, 2% glutamine, and 75% NaHCO_3_). Blood stage parasites in the ring form obtained in sorbitol-synchronized blood (180 *μ*L/well) were cultured in 96-well culture plates at 1% parasitemia and 1% hematocrit and then incubated with the extracts. Controls without extracts or with chloroquine, used as the reference antimalarial drug, were run in parallel. After a 24 h incubation period, 20 *μ*L of medium containing [^3^H]-hypoxanthine (0.5 *μ*Ci/well) was added to each well, followed by incubation for 18 h at 37°C. The plates were frozen and thawed, and the cells were harvested (Tomtec 96-Harvester; Tomtec Inc., Handem, CT, USA) on prewet glass-fiber filters (Wallac Ou, Turku, Finland), which were placed in sample bags (Wallac) and immersed in scintillation fluid (OptiPhase Super Mix, Wallac). Radioactive emission was counted in a 1450 Microbeta reader (Wallac). 

 The half-maximal inhibitory concentrations (IC_50_) as compared to the drug-free controls were estimated by using curve-fitting software (Microcal Origin Software 8.0, Inc.). Each experiment was repeated at least two times. The microtitre plates used for the radioactive assay were kept in a designated place and are meant to be there for a minimum of eighty years.

#### 2.7.2. Histidine-Rich Protein 2 (HRP2) Assay

This was performed according to the method of Noedl et al. [14]. The samples from the continuous cultures were washed and resuspended with RPMI 1640 (with 10% human serum) and uninfected erythrocytes to obtain 0.05% parasitemia and 1.5% hematocrit. For the test, a stock solution of each extract fraction was diluted in complete culture medium without hypoxanthine (RPMI 1640 containing 10% human serum, 2% glutamine, and 75% NaHCO_3_). Serial twofold dilutions (seven concentrations and one drug-free control) of the drugs (20 *μ*L/well) were dispensed into standard 96-well microtitre plates (Costar 3599 plates), and 180 *μ*L of CMM was added to each well. The plates were then incubated for 72 h at 37.5°C. They were subsequently frozen-thawed twice to obtain complete hemolysis. 100 *μ*L each of the hemolyzed culture samples was transferred to the ELISA plates, which are precoated with monoclonal antibodies against *P. falciparum *HRP2 (capture antibody of the immunoglobulin M class; code CPF4), and the plates were incubated at room temperature for 1 h. Subsequently, the plates were washed four times with the washing solution provided with the test kit, and 100 *μ*L of the diluted antibody conjugate (an indicator antibody of the immunoglobulin G1 isotype; code CPF6) was added to each well. After incubation for an additional 1 h, the plates were washed four times, and 100 *μ*L of diluted (1 : 20) chromogen (tetramethylbenzidine) was added to each well. The plates were then incubated for another 15 min in the dark, and 50 *μ*L of the stop solution was added. Spectrophotometric analysis was performed with an ELISA plate reader (SpectraMAX 340 Microplate spectrophotometer; Molecular Devices, Sunnyvale, CA, USA) at 450 nm. 

#### 2.7.3. Cytotoxicity Test Using Cultures of Hepatoma Cell Line

Hep G2 A16 hepatoma cells were kept at 37°C in RPMI supplemented with 5% fetal calf serum (complete medium), in a 5% CO_2_ environment. Cells from confluent monolayers were trypsinized, washed, counted, resuspended in complete medium, distributed in 96-well microtiter plates (4 × 10^4^ cells/well), and then incubated for another 18 h at 37°C. The extract fractions prepared as stock solutions in DMSO were diluted in incomplete RPMI without fetal calf serum. After 24 h incubation at 37°C, 20 *μ*L of MTT solution (5 mg/mL in RPMI 1640 without phenol red) was added to each well [15]. After 3 h incubation at 37°C, the supernatant was removed, and 100 *μ*L of dimethyl sulphoxide was added to each well. The culture plates were read using a spectrophotometer with a 570 nm filter and a background of 630 nm. The minimum lethal dose (MLD) that killed 50% of the cells was determined as reported by do Ceu de Madureira et al. [16]; each assay was performed two times at least. Based on the values of cytotoxicity and antimalarial activity the selectivity index of activity, (SI) was calculated using the formula SI = MLD_50_/IC_50_.

### 2.8. *In Vivo* Studies

#### 2.8.1. Animal Handling

The animals were housed in standard plastic cages and acclimatized for a period of two weeks. They were maintained under standard conditions (12 h light and 12 h dark cycle) and had access to chow (Bendel Feeds, Ewu, Edo State, Nigeria) and clean tap water *ad libitum*.

#### 2.8.2. 4-Day Suppressive Test in Animal Model

The antimalarial tests were performed using *P. berghei* NK65 strain, maintained by serial weekly passages of infected blood in mice. Tests were performed as described by Peters [17] with some modifications [18]. Briefly, thirty-five mice inoculated by intraperitoneal route with 1 × 10^5^ infected red blood cells were kept together for 2 to 16 h, divided randomly in seven groups of 5 mice per cage, and then treated daily by oral route for 3 consecutive days with various doses of the active extract fraction as follows.

 Group A (control): administered appropriate volume of 5% DMSO.
 Group B: administered 31.25 mg/Kg body weight of WAT ethyl acetate extract fraction.
 Group C: administered 62.5 mg/Kg body weight of WAT ethyl acetate extract fraction.
 Group D: administered 125 mg/Kg body weight of WAT ethyl acetate extract fraction.
 Group E: administered 250 mg/Kg body weight of WAT ethyl acetate extract fraction.
 Group F: administered 500 mg/Kg body weight of WAT ethyl acetate extract fraction.
 Group G: administered 20 mg/Kg body weight of chloroquine

All solutions were freshly prepared before administration. The extract fraction was then dissolved in 5% DMSO and then diluted so that each mouse received 200 *μ*L. At several days after parasite inoculation, blood smears were prepared from each mouse tail, methanol-fixed, stained with Giemsa, and then microscopically examined by counting parasitemia in up to 6000 erythrocytes. Inhibition of parasite growth in the drug-treated groups was calculated in relation to the nontreated control mice. The cumulative mortality of the animals was daily monitored up to day 30 after inoculation. 

#### 2.8.3. Toxicity Test in Animal Model

*Animal Grouping and Extract Administration*. Thirty adult Swiss mice were randomly divided into six groups of five mice each and daily administered 200 *μ*L of the various doses of most active extract fraction (WAT ethyl acetate extract fraction) dissolved in 5% DMSO by the oral route for 7 days as follows.

 Group A (control): administered 0.2 mL 5% DMSO.
 Group B: administered 31.25 mg/Kg body weight of WAT ethyl acetate extract fraction.
 Group C: administered 62.5 mg/Kg body weight of WAT ethyl acetate extract fraction.
 Group D: administered 125 mg/Kg body weight of WAT ethyl acetate extract fraction.
 Group E: administered 250 mg/Kg body weight of WAT ethyl acetate extract fraction.
 Group F: administered 500 mg/Kg body weight of WAT ethyl acetate extract fraction.

*Sample Collection and Preparation*. At the end of the 7-day experimental period, the mice were sacrificed by slight diethyl ether anaesthesia, and venous blood was collected into EDTA bottle to prevent clotting. The EDTA blood sample was centrifuged at 3000 rpm for 5 min and the plasma pipetted out. This was stored frozen until needed for analysis. 

#### 2.8.4. Biochemical Assays

The protein content of the plasma was determined using the Biuret method as reported by Gornall et al. [19]. The plasma creatinine and urea concentrations were determined by the methods of Bartels and Bohmer [20] and Veniamin and Vakirtzi-Lemonias [21], respectively. Bilirubin concentration in the plasma was determined using the method described by Winsten and Cehely [22]. The procedure described by Doumas et al. [23] was used for the determination of plasma albumin concentration of the mice. The determination of plasma globulin concentration was done using the method described by Tietz [24] by subtracting the concentrations of liver and plasma albumin from the total liver and plasma protein concentrations, respectively. Alkaline phosphatase activity was determined by the method of Wright et al. [25]. Aspartate aminotransferase (AST) and alanine aminotransferase (ALT) activities were determined by monitoring the concentrations of *α*-keto acid hydrazones formed with 2, 4-dinitrophenyl hydrazine based on the procedure of Reitman and Frankel [26]. The method described by Wroblewski and La due [27], with slight modification, was used for the determination of lactate dehydrogenase activity. The method described by Bradley et al. [28], with slight modification, was used for the determination of glutamate dehydrogenase activity.

### 2.9. Statistical Analysis

Data were analyzed using Duncan multiple range test following one-way analysis of variance (ANOVA) using SPSS 16.0 computer software package (SPSS Inc., Chicago, IL, USA). Differences at *P* < 0.05 were considered significant.

## 3. Results

The *in vitro* evaluation of the extract fractions for antiplasmodial activities revealed that only the ethyl acetate fraction of the husk fibre extract of WAT was active against *Plasmodium falciparum* W2 strain maintained in continuous culture (IC_50_ = 10.94 *μ*g/mL), with a selectivity index of 30.3 (Table 1). Only extracts with IC_50_ less than 25 *μ*g/mL are generally considered active [18]. The phytochemicals present in the WAT ethyl acetate extract fraction were found to be alkaloids, tannins, and flavonoids (Table 2). The 4-day suppressive test revealed that the WAT ethyl acetate extract fraction, at the doses of 31.25, 62.5, and 125 mg/Kg body weight, caused 50.2%, 97.5%, and 98.6% reduction in parasitemia, respectively, on day 4 after inoculation and 56.6%, 73.4%, and 71.1% reduction in parasitemia, respectively, on day 6 after inoculation (Table 3). There was no significant change (*P* > 0.05) in plasma urea concentration caused by the extract fraction at all doses administered whereas the extract fraction significantly increased (*P* < 0.05) plasma creatinine concentration at the doses of 250 and 500 mg/Kg body weight compared to controls (Table 4). Administration of WAT ethyl acetate extract fraction, at all doses investigated, did not significantly (*P* > 0.05) alter the plasma total and conjugated bilirubin concentrations (Table 5), as well as plasma albumin and globulin compared to controls (Table 6). All doses of the extract fraction administered caused no significant alteration (*P* > 0.05) in plasma total cholesterol concentration, HDL cholesterol concentration, and atherogenic index compared to controls (Table 7). However, the extract fraction significantly increased (*P* < 0.05) the plasma triglyceride concentration at the dose of 31.25 mg/kg body weight compared to control. Moreover, WAT ethyl acetate extract fraction, at all doses administered, did not significantly alter (*P* > 0.05) plasma alkaline phosphatase and alanine aminotransferase activities but significantly reduced (*P* < 0.05) plasma aspartate aminotransferase activity compared to controls (Table 8). However, plasma lactate dehydrogenase activity was increased significantly (*P* < 0.05) at the dose of 250 mg/kg body weight and glutamate dehydrogenase activity at the doses of 62.5 and 250 mg/Kg body weight compared to controls (Table 9).

## 4. Discussion

*Cocos nucifera* is abundant in Nigeria and other parts of West Africa [29]. The plant husk fibre has been reported to be used in the middle belt region of Nigeria as an antimalarial remedy, which has also been authenticated through *in vitro* studies [8]. The results of this study lend credence to our earlier report on the antiplasmodial activity of the widely grown tall variety of *Cocos nucifera* [8] and also established the *in vivo* antimalarial activity of the active extract fraction, WAT ethyl acetate extract fraction. Phytochemical screening of WAT ethyl acetate extract fraction in this study revealed the presence of flavonoids, tannins, and alkaloids (Table 2). This result is similar to the report of Silva et al. [30] on the phytochemicals present in the ethyl acetate extract of *Cocos nucifera* (Olho de Cravo variety) husk fibre. Moreover, tannins and polyphenolic compounds such as flavonoids, of which catechins are the most prominent, have been reported to be present in abundance in the coconut husk fibre and are responsible for its antibacterial, antiviral, antileishmanial, antinociceptive and, free radical scavenging activities [31, 32]. Flavonoids and tannins present, which are phenolic compounds, act primarily as antioxidants or free radical scavengers, and may alleviate the oxidative stress associated with malaria, which plays an important role in the development of anaemia in malaria [33].

The concentrations of total protein, bilirubin, and albumin in blood can be used to ascertain different types of liver damage [34]. Bilirubin is an important product of haemoglobin catabolism with biological and diagnostic values [35]. Absence of a significant (*P* > 0.05) change in both total and conjugated bilirubin suggests that the extract fraction, at the doses administered in this study, is not capable of causing haemolysis and impairment of the secretion of conjugated bilirubin into the bile duct in the liver [36].

Albumin is synthesized in the liver. Thus, the lack of alteration in plasma albumin concentration suggests that the synthetic function of the liver has not been compromised, at all doses of the extract fraction administered [37]. Globulins are heterogeneous complex mixture of protein molecules with diverse functions in the body [38]. Also, there was no significant change in the globulin concentrations in plasma caused by the extract fraction at the doses administered in this study. Thus, the roles of these plasma proteins in the transportation of nutrients, defense mechanism, coagulation processes, maintenance of blood osmotic pressure, and buffering capacity of the blood may not be affected by the extract fraction [38].

Serum urea and creatinine concentrations are used for the assessment of renal sufficiency. Higher than normal levels serum urea and creatinine are indicators of deficiency in renal function [39]. Moreover, increase in serum urea level may also be due to the increase in protein catabolism [40] while the decreased urea level may be attributed to impairment of the urea cycle leading to reduced production of urea [9]. The extract fraction, at all doses administered, showed no significant alteration in the plasma urea concentration, suggesting that the urea cycle and protein catabolism were not adversely affected by the extract fraction. However, the increase in plasma creatinine concentration at the doses of 250 and 500 mg/Kg body weight suggests that the extract fraction may impair renal function at higher doses. 

In recent times, there has been an increase in the prevalence of coronary heart disease (CHD) and CHD-related deaths possibly due to the mismanagement of the risk factors that predispose to this disorder [41]. The major identified risk factors are elevated serum LDL-cholesterol concentration, reduced serum HDL-cholesterol concentration, and high blood pressure [42, 43]. Studies have shown that lowering levels of serum cholesterol decreases the incidence of coronary heart diseases [44]. The atherogenic index (total cholesterol/HDL-cholesterol) is a reliable and strong indicator of cardiovascular diseases. Myocardial infarction increases considerably when the ratio is higher than 5 [45, 46]. All the values for atherogenic index observed in this study were less than 3 and were not significantly different (*P* > 0.05) from control, suggesting that WAT ethyl acetate extract fraction may not predispose subjects to coronary heart disease, thereby not giving rise to further cardiovascular complications during malaria treatment.

The measurement of the activities of enzymes in tissues and body fluids plays a paramount and well-known role in disease investigation and diagnosis [47]. These enzymes, such as phosphatases, dehydrogenases, and transferases, get into the blood through leakage from disrupted cell membranes in damaged tissues [48, 49]. Alkaline phosphatase (ALP) has been reported to be a marker enzyme for plasma membrane and endoplasmic reticulum [50]. ALT activity in the plasma is a more specific indicator of liver damage affecting cell integrity [51]. ALP and ALT activities, at all doses of the extract fraction administered, were not significantly changed in the plasma, suggesting that the extract fraction may not interfere with plasma membrane integrity and other metabolic activities mediated by ALP in the liver [52]. Lack of change in plasma ALP activity also suggests that the extract fraction does to cause hepatobiliary obstruction [51]. The reduction in plasma AST activity, at all doses of the extract fraction administered in this study, suggests inhibition of the enzyme *in situ* rather than damage to the liver or heart. Generally, plasma lactate dehydrogenase activity was not affected by the extract fraction, except at the dose of 250 mg/kg body weight, suggesting that the extract fraction not predispose subjects to myocardial infarction and haemolysis [51].

In conclusion, the results of this study suggest the husk fibre of the West African Tall variety of *Cocos nucifera* as a potential source for novel antimalarial drug, pinpointing its ethyl acetate extract fraction as being responsible for its antimalarial activity. This active extract fraction may not possess hepatotoxicity potential nor predispose subjects to cardiovascular diseases. However, it may impair normal kidney function at higher doses. Further studies on biofractionation are underway to isolate the active compound(s) from the active extract fraction, with the aim of getting rid of the nephrotoxicity of the extract fraction at higher doses and reducing the expensive cost of the treatment of the disease, especially amidst the poor populace of the country.

## Figures and Tables

**Table 1:** Antiplasmodial activities and cytotoxicities of fractions of *Cocos nucifera* husk fibre extracts.

Extracts	IC_50_ (*µ*g/mL) *Plasmodium falciparum *W2			Remark	MLD_50_ (*µ*g/mL)*	Selectivity index
	Hypoxanthine	HRP2	Mean			
DG (H)	>50.00	>50.00	>50.00	Inactive	>1000.00 **±** 0.00	—
DG (M)	—	>50.00	>50.00	Inactive	>1000.00 **±** 0.00	—
DR (EA)	>50.00	>50.00	>50	Inactive	452.50 **±** 136.50	—
DR (H)	43.00	>50.00	>50.00	Inactive	>1000.00 **±** 0.00	—
DR (M)	>50.00	>50.00	>50.00	Inactive	>1000.00 **±** 0.00	—
DY (EA)	9.80	>50.00	—	Inconclusive	>1000.00 **±** 0.00	—
DY (M)	>50.00	>50.00	>50.00	Inactive	635.70 **±** 48.50	—
DY (H)	>50.00	>50.00	>50.00	Inactive	>1000.00 **±** 0.00	—
HB (EA)	>50.00	>50.00	>50.00	Inactive	332.70 **±** 33.00	—
HB (H)	>50.00	>50.00	>50.00	Inactive	>1000.00 **±** 0.00	—
HB (M)	>50.00	26.70	—	Inactive	>1000.00 **±** 0.00	—
WAT (EA)	12.44	9.43	10.94 ± 2.00	Active	333.00 **±** 25.50	30.30
WAT (H)	46.60	30.50	39.00 ± 8.00	Inactive	408.00 **±** 42.40	—
WAT (M)	14.60	>50.00	—	Inconclusive	>1000.00 **±** 0.00	—
Chloroquine	0.04	0.05	0.04 ± 0.02	Active	387.50 ± 47.50	9687.50

*Mean of 3 experiments ± SD. WAT: West African Tall variety; DG: Dwarf Green variety; DR: Dwarf Red variety; DY: Dwarf Yellow variety; HB: Hybrid variety; M: methanolic fraction; H: hexane fraction; EA: ethyl acetate fraction.

**Table 2:** Phytochemicals of ethyl acetate fraction of *Cocos nucifera* (West African Tall variety) husk fibre extract.

Phytochemical	Status in extract fraction
Tannins	+
Saponins	ND
Alkaloids	+
Phlobatannins	ND
Glycosides	ND
Steroids	ND
Flavonoids	+
Phenols	ND

+: present; ND: not detected.

**Table 3:** Parasitaemia in *P. berghei* NK65-infected mice treated with ethyl acetate fraction of *Cocos nucifera* (West African Tall variety) husk fibre extract.

Groups (dose in mg/kg b.w.)	Parasitaemia (% reduction)			
	4°	6°	8°	10°
Untreated control	2.85	5.71	6.43	9.48
31.25	1.42 (50.2)	2.48 (56.6)	5.20 (19.1)	7.80 (17.7)
62.5	0.07 (97.5)	1.52 (73.4)	0.75 (88.3)	3.58 (62.2)
125	0.04 (98.6)	1.65 (71.1)	0.88 (86.3)	2.49 (73.7)
250	2.02 (29.1)	2.00 (64.9)	1.52 (76.4)	1.60 (83.1)
500	0.37 (87.0)	0.68 (88.1)	0.15 (97.7)	1.21 (87.2)
Chloroquine (20)	0.04 (98.6)	1.23 (78.5)	0.65 (89.95)	0.23 (97.6)

Values are means ± SD of 5 replicates. °Days after inoculation.

**Table 4:** Effects of ethyl acetate fraction extract of *Cocos nucifera* (West African Tall variety) husk fibre extract on plasma urea and creatinine concentrations in mouse.

Groups (dose in mg/kg b.w.)	Urea (mmol/L)	Creatinine (mmol/L)
5% DMSO (control)	7.63 ± 0.73^a^	75.62 ± 5.67^a^
31.25	6.32 ± 0.50^a^	88.70 ± 4.29^a^
62.5	6.16 ± 1.15^a^	83.98 ± 1.52^a^
125	6.39 ± 0.15^a^	87.62 ± 1.29^a^
250	6.16 ± 0.39^a^	115.77 ± 6.52^b^
500	7.28 ± 0.90^a^	115.03 ± 2.92^b^

Values are expressed as mean ± SEM (*n* = 5). Values in the same column with different superscripts are significantly different (*P* < 0.05).

**Table 5:** Effect of ethyl acetate fraction of *Cocos nucifera* (West African Tall variety) husk fibre extract on total and conjugated bilirubin concentrations in mouse plasma.

Groups (dose in mg/kg b.w.)	Total bilirubin (*μ*mol/L)	Conjugated bilirubin (*µ*mol/L)
5% DMSO (control)	333.86 ± 97.53^a^	113.58 ± 21.94^a^
31.25	471.13 ± 119.26^a^	121.23 ± 41.88^a^
62.5	568.98 ± 126.14^a^	128.23 ± 34.50^a^
125	371.99 ± 65.24^a^	155.88 ± 42.59^a^
250	467.15 ± 85.70^a^	151.54 ± 42.45^a^
500	370.50 ± 139.87^a^	103.13 ± 25.09^a^

Values are mean ± SEM of 5 replicates. Values in the same column with same letter superscripts are not significantly different (*P* > 0.05).

**Table 6:** Effects of ethyl acetate fraction of *Cocos nucifera* (West African Tall variety) husk fibre extract on total protein, albumin, and globulin concentrations in mouse plasma.

Groups (dose in mg/kg b.w.)	Total protein (mg/mL)	Albumin (g/L)	Globulin (g/L)
5% DMSO (control)	22.64 ± 0.86^b^	27.86 ± 1.59^a^	5.86 ± 0.44^ab^
31.25	24.84 ± 1.03^b^	30.09 ± 1.57^a^	6.35 ± 1.96^ab^
62.5	26.19 ± 2.04^b^	25.25 ± 1.95^a^	1.98 ± 0.57^a^
125	21.76 ± 1.05^b^	25.58 ± 1.74^a^	5.66 ± 1.12^ab^
250	16.46 ± 2.31^a^	24.95 ± 0.93^a^	8.50 ± 3.02^b^
500	21.41 ± 1.32^b^	27.61 ± 1.01^a^	6.20 ± 0.38^ab^

Values are mean ± SEM of 5 replicates. Values in the same column with different letter superscripts are significantly different (*P* < 0.05).

**Table 7:** Effects of ethyl acetate fraction of *Cocos nucifera *(West African Tall variety) husk fibre extract on plasma lipid profile.

Groups (dose in mg/kg b.w.)	Chol (mmol/L)	HDL-chol (mmol/L)	TG (mmol/L)	Atherogenic index
5% DMSO (control)	2.11 ± 0.36^a^	1.01 ± 0.32^a^	0.91 ± 0.15^a^	2.09 ± 0.06^a^
31.25	1.96 ± 1.43^a^	0.90 ± 0.22^a^	1.50 ± 0.41^b^	2.18 ± 0.32^a^
62.5	1.48 ± 0.89^a^	0.71 ± 0.12^a^	1.18 ± 0.18^ab^	2.11 ± 0.22^a^
125	1.71 ± 1.29^a^	0.82 ± 0.32^a^	1.01 ± 0.22^a^	2.10 ± 0.36^a^
250	2.84 ± 1.5^a^	1.25 ± 0.33^a^	1.09 ± 0.28^a^	2.62 ± 0.41^a^
500	1.50 ± 0.89^a^	0.85 ± 0.27^a^	0.91 ± 0.60^a^	1.15 ± 0.04^a^

Values are means ± SEM, *n* = 5. Values in each column with different letter superscripts are significantly different (*P* < 0.05).

**Table 8:** Effects of ethyl acetate fraction of *Cocos nucifera *(West African Tall variety) husk fibre extract on mouse plasma alkaline phosphatase, aspartate, and alanine aminotransferases activities.

Groups (dose in mg/kg b.w.)	ALP (*μ*mol/mg prot./min)	ALT (mmol pyr/min/mg prot.)	AST (×10^−5^ mmol pyr/min/mg prot.)
5% DMSO (control)	2.49 ± 0.36^a^	81.20 ± 17.05^a^	20.57 ± 0.30^e^
31.25	3.25 ± 0.18^a^	159.39 ± 50.31^a^	4.47 ± 0.48^b^
62.5	2.74 ± 0.22^a^	122.83 ± 35.09^a^	7.50 ± 1.00^c^
125	2.79 ± 0.17^a^	222.50 ± 35.32^a^	7.93 ± 0.24^c^
250	2.75 ± 0.20^a^	151.83 ± 22.59^a^	0.60 ± 0.01^a^
500	2.77 ± 0.39^a ^	154.74 ± 11.76^a^	15.00 ± 1.00^d^

Values are mean ± SEM of 5 replicates. Values with different letter superscripts from the control are significantly different (*P* < 0.05).

**Table 9:** Effects of ethyl acetate fraction of *Cocos nucifera *(West African Tall variety) husk fibre extract on activities of mouse plasma lactate and glutamate dehydrogenases.

Groups (dose in mg/kg b.w.)	Lactate dehydrogenase (*μ*M/mg protein/min)	Glutamate dehydrogenase (U/mg protein)
5% DMSO (control)	0.11 ± 0.01^a^	0.08 ± 0.01^a^
31.25	0.13 ± 0.01^a^	0.09 ± 0.02^a^
62.5	0.12 ± 0.01^a^	0.36 ± 0.07^b^
125	0.14 ± 0.01^a^	0.04 ± 0.010^a^
250	0.19 ± 0.03^b^	0.25 ± 0.06^b^
500	0.13 ± 0.01^a ^	0.07 ± 0.01^a ^

Values are mean ± SEM of 5 replicates. Values with different letter superscripts from the control are significantly different (*P* < 0.05).

## References

[1] WHO (2011). *World Malaria Report 2011*.

[2] WHO (2008). *World Malaria Report 2008*.

[3] Adebayo JO, Krettli AU (2011). Potential antimalarials from Nigerian plants: a review. *Journal of Ethnopharmacology*.

[4] WHO (2010). *World Malaria Report 2010*.

[5] Sachs J, Malaney P (2002). The economic and social burden of malaria. *Nature*.

[6] Willcox ML (1999). A clinical trial of “AM”, a Ugandan herbal remedy for malaria. *Journal of Public Health Medicine*.

[7] Al-Adhroey AH, Nor ZM, Al-Mekhlafi HM, Amran AA, Mahmud R (2011). Evaluation of the use of *Cocos nucifera* as antimalarial remedy in Malaysian folk medicine. *Journal of Ethnopharmacology*.

[8] Adebayo JO, Santana AEG, Krettli AU (2012). Evaluation of the antiplasmodial and cytotoxicity potentials of husk fiber extracts from *Cocos nucifera*, a medicinal plant used in Nigeria to treat human malaria. *Human and Experimental Toxicology*.

[9] Adebayo JO, Yakubu MT, Egwim EC, Owoyele VB, Enaibe BU (2003). Effect of ethanolic extract of Khaya senegalensis on some biochemical parameters of rat kidney. *Journal of Ethnopharmacology*.

[10] Sofowora A (1993). *Medicinal Plants and Traditional Medicine in Africa*.

[11] Trager W, Jensen JB (1976). Human malaria parasites in continuous culture. *Science*.

[12] Desjardins RE, Canfield CJ, Haynes JD, Chulay JD (1979). Quantitative assessment of antimalarial activity *in vitro* by a semiautomated microdilution technique. *Antimicrobial Agents and Chemotherapy*.

[13] Zalis MG, Pang L, Silveira MS, Milhous WK, Wirth DF (1998). Characterization of *Plasmodium falciparum* isolated from the Amazon region of Brazil: evidence for quinine resistance. *The American Journal of Tropical Medicine and Hygiene*.

[14] Noedl H, Wernsdorfer WH, Miller RS, Wongsrichanalai C (2002). Histidine-rich protein II: a novel approach to malaria drug sensitivity testing. *Antimicrobial Agents and Chemotherapy*.

[15] Denizot F, Lang R (1986). Rapid colorimetric assay for cell growth and survival—modifications to the tetrazolium dye procedure giving improved sensitivity and reliability. *Journal of Immunological Methods*.

[16] do Ceu de Madureira M, Martins AP, Gomes M, Paiva J, da Cunhaa AP, do Rosario V (2002). Antimalarial activity of medicinal plants used in traditional medicine in S. Tome and Prıncipe Island. *Journal of Ethnopharmacology*.

[17] Peters W (1965). Drug resistance in *Plasmodium berghei* Vincke and Lips, 1948. I. Chloroquine resistance. *Experimental Parasitology*.

[18] Carvalho LH, Brandão MGL, Santos-Filho D, Lopes JLC, Krettli AU (1991). Antimalarial activity of crude extracts from Brazilian plants studied *in vivo* in *Plasmodium berghei*-infected mice and *in vitro* against *Plasmodium falciparum* in culture. *Brazilian Journal of Medical and Biological Research*.

[19] Gornall AG, Bardawill CJ, David MM (1949). Determination of serum proteins by means of the biuret reaction. *The Journal of Biological Chemistry*.

[20] Bartels H, Bohmer M (1972). Kinetic determination of creatinine concentration. *Clinica Chimica Acta*.

[21] Veniamin MP, Vakirtzi-Lemonias C (1970). Chemical basis of the carbamidodiacetyl micromethod for estimation of urea, citrulline, and carbamyl derivatives. *Clinical Chemistry*.

[22] Winsten S, Cehely KB (1968). *Clinical Chemistry*.

[23] Doumas BT, Watson WA, Biggs HG (1971). Albumin standards and the measurement of serum albumin with bromcresol green. *Clinica Chimica Acta*.

[24] Tietz NW (1995). *Clinical Guide to Laboratory Tests*.

[25] Wright PJ, Leathwood PD, Plummer DT (1972). Enzymes in rat urine: alkaline phosphatase. *Enzymologia*.

[26] Reitman S, Frankel S (1957). A colorimetric method for the determination of serum glutamic oxalacetic and glutamic pyruvic transaminases. *The American Journal of Clinical Pathology*.

[27] Wroblewski F, La Due JS (1955). Lactate dehydrogenase activity in blood. *Proceedings of the Society for Experimental Biology and Medicine*.

[28] Bradley BA, Colen AH, Fisher HF (1979). The effects of methanol on the glutamate dehydrogenase reaction at 0°C. *Biophysical Journal*.

[29] Shukla A, Mehrotra RC, Guleria JS (2012). Cocos sahnii Kaul: a *Cocos nucifera* L. like fruit from the early eocene rainforest of Rajasthan, Western India. *Journal of Biosciences*.

[30] Silva LCR, Nunes-Pinheiro DCS, Morais SM, Lopes-Neto BE, Santos GJL, Campello CC (2009). Toxicological evaluation and effect of ethyl acetate extract of the fiber of *Cocos nucifera* L. (Palmae) on inflammatory response *in vivo*. *Revista Brasileira de Plantas Medicinais*.

[31] Esquenazi D, Wigg MD, Miranda MMFS (2002). Antimicrobial and antiviral activities of polyphenolics from *Cocos nucifera* Linn. (Palmae) husk fiber extract. *Research in Microbiology*.

[32] Mendonça-Filho RR, Rodrigues IA, Alviano DS (2004). Leishmanicidal activity of polyphenolic-rich extract from husk fiber of *Cocos nucifera* Linn. (Palmae). *Research in Microbiology*.

[33] Ayoola GA, Coker HAB, Adesegun SA (2008). Phytochemical screening and antioxidant activities of some selected medicinal plants used for malaria therapy in Southwestern Nigeria. *Tropical Journal of Pharmaceutical Research*.

[34] Naganna B, Tawalar GP, Srivastava LM, Moudgils KD (1989). Plasma proteins. *Textbook of Biochemistry and Human Biology*.

[35] Hopkins PN, Wu LL, Hunt SC, James BC, Vincent GM, Williams RR (1996). Higher serum bilirubin is associated with decreased risk for early familial coronary artery disease. *Arteriosclerosis, Thrombosis, and Vascular Biology*.

[36] Warrel DA, Francis N, McIntyre N, Benhamou UP, Bircher J, Rizzetto M, Robes J (1992). Malaria. *Oxford Textbook of Clinical Hepatology*.

[37] Nicholson JP, Wolmarans MR, Park GR (2000). The role of albumin in critical illness. *The British Journal of Anaesthesia*.

[38] Ganong WF (2000). Circumventricular organs: definition and role in the regulation of endocrine and autonomic function. *Clinical and Experimental Pharmacology and Physiology*.

[39] Oyewole OI, Oladipupo OT, Atoyebi BV (2012). Assessment of renal and hepatic functions in rats administered methanolic leaf extract of *Jatropha tanjorensis*. *Annals of Biological Research*.

[40] Whealton A, Watson AJ, Rock RC, Burtis CA, Ashwood ER (1994). *Tietz Textbook of Clinical Chemistry*.

[41] WHO (2009). Cardiovascular diseases. *Fact Sheet No.*.

[42] Stamler J, Wentworth D, Neaton JD (1986). Is relationship between serum cholesterol and risk of premature death from coronary heart disease continuous and graded? Findings in 356,222 primary screenees of the multiple risk factor intervention trial (MRFIT). *The Journal of the American Medical Association*.

[43] Kannel WB (1987). New perspectives on cardiovascular risk factors. *The American Heart Journal*.

[44] Steiner M, Li W (2001). Aged garlic extract, a modulator of cardiovascular risk factors: a dose-finding study on the effects of AGE on platelet functions. *Journal of Nutrition*.

[45] Boers M, Nurmohamed MT, Doelman CJA (2003). Influence of glucocorticoids and disease activity on total and high density lipoprotein cholesterol in patients with rheumatoid arthritis. *Annals of the Rheumatic Diseases*.

[46] Castelli WP (1998). The new pathophysiology of coronary artery disease. *The American Journal of Cardiology*.

[47] Malomo SO (2000). Toxicological implications of ceftriaxone administration in rats. *Nigerian Journal of Biochemistry and Molecular Biology*.

[48] Coodley EL, Coodley EL (1970). *Diagnostic Enzymologia*.

[49] Adaramoye OA, Osaimoje DO, Akinsanya AM, Nneji CM, Fafunso MA, Ademowo OG (2008). Changes in antioxidant status and biochemical indices after acute administration of artemether, artemether-lumefantrine and halofantrine in rats. *Basic and Clinical Pharmacology and Toxicology*.

[50] Wright PJ, Plummer DT (1974). The use of urinary enzyme measurements to detect renal damage caused by nephrotoxic compounds. *Biochemical Pharmacology*.

[51] Panteghini M, Bais R, Burtis CA, Ashwood ER, Border BG, Tietz NW (2008). Enzymes. *Tietz Fundamentals of Clinical Chemistry*.

[52] Umezawa H, Hooper IR (1982). *Amino-Glycoside Antibiotic*.

